# Do street-level scene perceptions affect housing prices in Chinese megacities? An analysis using open access datasets and deep learning

**DOI:** 10.1371/journal.pone.0217505

**Published:** 2019-05-30

**Authors:** Xiao Fu, Tianxia Jia, Xueqi Zhang, Shanlin Li, Yonglin Zhang

**Affiliations:** 1 State Key Laboratory of Urban and Regional Ecology, Research Center for Eco-Environmental Sciences, Chinese Academy of Sciences, Beijing, China; 2 College of Resources and Environment, University of Chinese Academy of Sciences, Beijing, China; 3 National Science Library, Chinese Academy of Sciences, Beijing, China; Xiamen University, CHINA

## Abstract

Many studies have explored the relationship between housing prices and environmental characteristics using the hedonic price model (HPM). However, few studies have deeply examined the impact of scene perception near residential units on housing prices. This article used house purchasing records from FANG.com and open access geolocation data (including massive street view pictures, point of interest (POI) data and road network data) and proposed a framework named “open-access-dataset-based hedonic price modeling (OADB-HPM)” for comprehensive analysis in Beijing and Shanghai, China. A state-of-the-art deep learning framework and massive Baidu street view panoramas were employed to visualize and quantify three major scene perception characteristics (greenery, sky and building view indexes, abbreviated GVI, SVI and BVI, respectively) at the street level. Then, the newly introduced scene perception characteristics were combined with other traditional characteristics in the HPM to calculate marginal prices, and the results for Beijing and Shanghai were explored and compared. The empirical results showed that the greenery and sky perceptual elements at the property level can significantly increase the housing price in Beijing (RMB 39,377 and 6011, respectively) and Shanghai (RMB 21,689 and 2763, respectively), indicating an objectively higher willingness by buyers to pay for houses that provide the ability to perceive natural elements in the surrounding environment. This study developed quantification tools to help decision makers and planners understand and analyze the interaction between residents and urban scene components.

## Introduction

Urban scenes can be considered a vital medium for human beings, and it is important to recognize and understand this aspect of cities [1] to ensure the spatial security of urban sustainability [2]. The spatial distributions of urban scenes exhibit variety, complexity and heterogeneity [3], and this variability is necessary for urban residents to survive and thrive [4]. The recognition, judgment, understanding and feelings associated with urban perception can impact people’s daily lives [5]. Therefore, urban perception is closely related to the amenities of the urban visual environment. For instance, residential parcels filled with rubbish and painted with graffiti can make pedestrians feel unsafe and further negatively affect residents’ willingness to live in the area [6,7]. Some studies have shown that the perceptual elements in the urban environment have a vital influence on people’s mental and physical health [8–10]. Visible greenery can reduce pedestrians’ stress and passive emotions [11] and can soothe hospital patients and shorten their recovery time [12]. Places such as urban green parks and open plazas can provide green landscapes and necessary spaces for residents and pedestrians to perform various physical activities [13], which can lower the risks of heart disease, diabetes and obesity [14] and improve people’s physical fitness [15]. Recent research has demonstrated that street-level visible greenery can significantly improve people’s likelihood to walk [16,17]. Although high-density high-rise buildings can enhance the oppressiveness experienced by pedestrians, green trees planted along roads can eliminate this negative effect [8,9]. In addition, recent studies have suggested that the perception of the thermal environment is connected to the openness of street canyons [18], which have an impact on both ground surface temperature and pedestrians’ walking comfort and physical health [18,19]. In summary, thoroughly studying and quantifying human-level urban environmental perceptions can help promote urban sustainability and public health.

Many studies have shown that the neighborhood, location, and structure characteristics in residential environments influence both peoples’ willingness to pay and housing prices [20–22]. Neighborhood characteristics can be described as the accessibility of important urban infrastructure facilities (such as street greenery, subway stations, urban parks, financial and educational facilities) [23–30]; location characteristics include distance by road from housing estates to the city center [31–34]; and structure characteristics generally include land area, building age, property management fees, the greening rate and the floor-area ratio of housing estates [32,35–38]. However, very few studies have considered the impacts of scene perceptions on housing prices.

Jim and Chen’s [22] study in Hong Kong, China, used a questionnaire method, and their results proved that the visual impacts of high-density high-rise residential areas have a negative impact on housing prices, but they did not specify which scene elements affected housing prices. A case study in the Fifth Ring Road area in Beijing, China, showed that street-level visible greenery has a significant impact on housing prices [38], but other scene elements and the effects in other Chinese megacities have not been fully discussed. Therefore, based on previous works, this study sought to comprehensively analyze and discuss the influence of urban scene perceptions on housing prices in two different Chinese megacities.

The hedonic price model (HPM) has been widely used as a tool to explore and analyze the impact of environmental characteristics on housing prices. The basic theory of the HPM is based on the relations between commodity price and attributes [39]. The attributes of public goods in a market economy dynamically influence commodity prices because of the variations in buyer demand [39]. Therefore, the HPM is a typical and classic method for quantifying and assessing the characteristics that impact commodity prices. The HPM has been adopted to assess the values and service qualities of nonmarket public goods, such as air and water quality [40,41], landscape aesthetics [42], and noise [20]. A case study in Shenzhen, China, by Wu et al. [43] employed open access point of interest (POI) data to measure the accessibility of urban infrastructures and explore their marginal effects in the housing price market. The study proved that POI datasets were promising data sources for determining housing price data. Many studies have already shown that POI data are a common, readily available and inexpensive open data source with great advantages due to their wide coverage and large data volumes. Moreover, POI data have been widely employed to study the spatial distributions of urban amenities, urban expansion boundaries and land use classification [43–45]. A study in Beijing, China, by Xiao et al. [46] used both crawled POI data and housing price records to build an HPM to explore the influence of the accessibility of urban amenities on housing prices. The results of this study provided tools to obtain open access data (housing price records and POI data) from web crawler scripts [46]. However, no literature currently utilizes HPMs to analyze the impacts of scene perceptions around housing estates on housing prices in Chinese megacities. Hence, in this study, we use a deep learning framework to parse the indicators of scene perceptions and introduce them to HPMs.

Because of the limitations of this technique, it has been very difficult to quantify scene perceptions at a large spatial scale. In recent years, given the advancements in machine learning, computer vision and deep learning methods and the increase in the accessibility of open access datasets, researchers are now able to perform automatic scene parsing using massive street view datasets by writing scripts and algorithms. Some companies, such as Google, Baidu and Tencent, have launched their own street view map service platforms, offering their street view resources to the public through application program interfaces (APIs), which provide new ways to solve scientific problems in rapidly expanding cities. Li et al. [47] first adopted massive Google Street View pictures and image segmentation algorithms to assess and map the street-level visible greenery in Hartford, Connecticut, USA, using an indicator defined as the green view index (GVI). That study explored the relationship between tree crowns and visible greenery at eye level, and the results showed that GVI was suitable for analyzing pedestrians’ perceptual patterns [47]. Combined with socioeconomic data, their further study discussed the equity issues of green perception in residential zones, and their results showed that wealthy residents were more likely to observe greenery than poor residents in Hartford [48]. Another study [49] performed in Jianye District, Nanjing, China, employed Baidu street view datasets to assess scene quality with multiple visual variables using image processing algorithms. The studies by Long and Liu [50] and Dong et al. [51] adopted image segmentation methods based on color model thresholding; however, their methods had difficulty extracting multiple visual elements in urban scenes. Gebru et al. [52] adopted 50 million Google Street View pictures in US cities and a deep-learning-based computer vision method to evaluate large-scale demographics, and their results showed that an automated system for monitoring demographics could effectively save labor costs and supplement near real-time scene data.

The current state-of-the-art method for extracting multiple scene elements utilizes pixel-level semantic segmentation to classify each pixel in massive street view pictures. Automatic Python scripts can be written to process massive street view pictures, and ArcGIS or other geospatial statistical platforms can be adopted to analyze and visualize the scene perception indicators. Common deep learning frameworks for urban scene parsing include SegNet [53] and PSPNet (pyramid scene parsing network) [54]. Several typical studies, including Gong et al. [55] and Liang et al. [56], used these deep learning frameworks. Li et al. [57] used Google Street View and mobile phone trajectory data to quantify streetscape variables and the Walk Score, and the results implied that both street greenery and openness were associated with human walking activities. Another study utilized a similar method that used the decrease in sky openness caused by trees along streets to quantify the shade provided by trees along streets in Boston [58]. This study provided tools to quantify the changes in urban climate. In addition, other studies developed machine learning models to extract visual elements from street view pictures to analyze and explore the spatial distribution and the relationship with the urban environment [18,59,60]. For instance, Zeng et al. [18] developed a k-means model to rapidly calculate a sky openness indicator and further used this index to analyze the relationship between the sky openness and the thermal environment in street canyons. Yin et al. [60] used an aggregated channel features (ACF) detection framework to recognize and investigate the number of pedestrians in several American cities. Liu et al. [59] used machine learning models to automatically assess the physical qualities of the urban environment in Beijing, China.

The diversity of categories and the precision of quantification have obviously increased throughout the development of deep learning frameworks. Zhang et al. [3] proposed the concept of a scene semantic tree, which used a deep learning method to quantify visible street-level information and adopted a hierarchical data structure to classify multiple scene semantic indexes. That study combined space syntax indexes to explore the relationship between scene perception and Hong Kong’s urban spatial form. Furthermore, Zhang et al. [61] combined the Place Pulse V2.0 dataset from Massachusetts Institute of Technology (MIT) and used a support vector machine (SVM) to analyze the relationship among six different scene perception scores (safe, lively, beautiful, wealthy, depressing and boring) and the scene semantic indexes in the central areas of Beijing and Shanghai.

In summary, this article employed multisource datasets, including FANG.com house purchasing records, the Baidu POI dataset, massive Baidu street view panoramas and AutoNavi road shapefiles, to quantify neighborhood, structure, location and scene perception characteristics. We adopted a state-of-the-art deep learning algorithm to quantify multiple scene perception indicators to represent the urban visual environment from a person’s perspective. Moreover, we built a general hedonic price modeling framework to analyze the factors that influence housing prices in Beijing and Shanghai, China, and contrasted the intercity model results. In this article, multiple scene perception indicators are first introduced into an HPM, the marginal prices of these characteristics are calculated, and the influences of the characteristics on housing prices are then discussed. The major goals of this study were to 1. provide an open-access-dataset-based hedonic price modeling (OADB-HPM) framework; 2. provide automatic tools for quantifying street-level scene perception based on PSPNet; 3. explore the influence of the residential-unit-level scene perception indicators (GVI; building view index, BVI; and sky view index, SVI) on housing prices; and 4. compare the model results and determine the common purchasing preferences of urban residents and house buyers in different megacities.

## Materials and methods

### Study area

**Fig 1:**
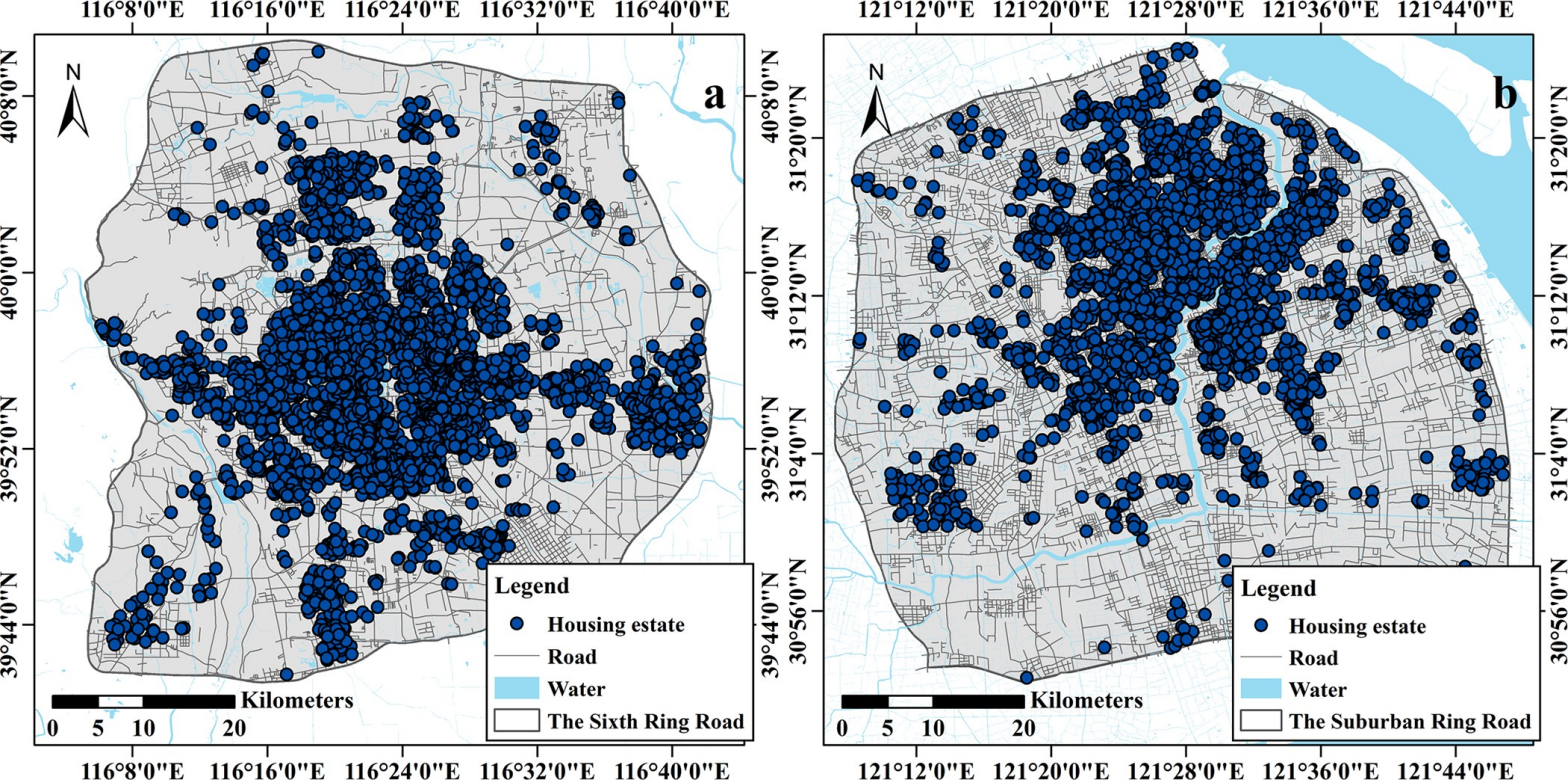
Study areas (the vectorized maps included the zone boundary, simplified road, water and housing plot shapefiles, and the corresponding files can be found at https://doi.org/10.6084/m9.figshare.7823969.v1).

Beijing and Shanghai are the two largest megacities in terms of financial, technological, economic, cultural and educational aspects on the Chinese mainland, and both have a certain influence on the international community. Approximately 15.34 million people resided within the Beijing Sixth Ring Road area (nearly 2265 km^2^) by 2015. The total road length in this area was approximately 5639 km, and the number of roads was approximately 13,008. In contrast, approximately 12.9 million people were living in the Shanghai suburban ring road area (nearly 2970 km^2^) by 2015. The total road length in this area was approximately 8466 km, and the number of road segments was approximately 21,892. The study areas in this paper are presented below in Fig 1 (subplot A is the sixth ring road area in Beijing, and subplot B is the suburban ring road in Shanghai). Beijing and Shanghai have remained first and second in terms of the volume and average price of real estate for decades. In addition, the housing prices in different localities in these two megacities highly differ; therefore, these cities are suitable for hedonic price modeling.

### The OADB-HPM framework

**Fig 2:**
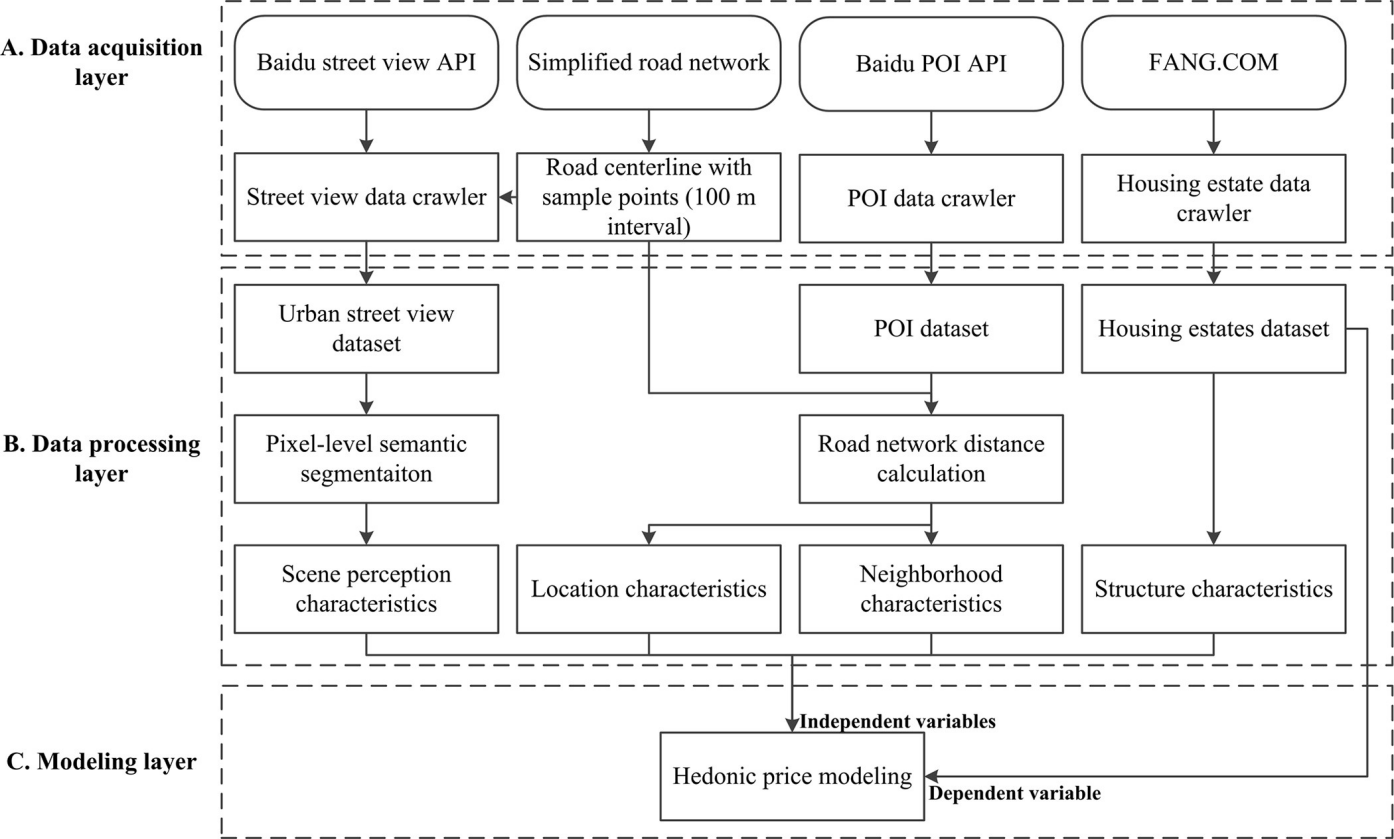
The open-access-dataset-based hedonic price modeling (OADB-HPM) framework.

This article introduces an open-access-dataset-based hedonic price modeling (OADB-HPM) framework (see Fig 2). The OADB-HPM framework includes three major processing layers: data acquisition, data processing and modeling layers. The whole framework is based on a top-down process that merges multisource open access datasets to construct an HPM. First, the data acquisition layer contains the data sources and acquisition tools. Second, the data processing layer includes the indicator calculation methods based on the multisource datasets obtained for the location, structure, neighborhood and scene perception characteristics. Finally, the main goal of the modeling layer is to construct an HPM based on the characteristics from the data processing layer and the housing prices at the plot level.

The multisource datasets in this article were composed of four parts: 1. Baidu street view panoramas of built-up areas in Beijing and Shanghai from March to October 2015; 2. simplified road shapefiles from AutoNavi in 2015 (https://doi.org/10.6084/m9.figshare.7823993.v1); 3. Baidu POI shapefiles, including the locations of bus and subway stations, restaurants, and medical, financial and education facilities (https://doi.org/10.6084/m9.figshare.7823969.v1); and 4. house purchasing records in 2015, which included data on the area, building age, households, orientation, greening ratio, floor-area ratio and property management fees (https://doi.org/10.6084/m9.figshare.7823972.v1). These four datasets offered data support to build HPMs for the central areas of Beijing and Shanghai. Before collecting the open source data, we double checked the terms of services for all relevant platforms to ensure that our study fully met the protocols.

The following paragraphs describe the layers of the OADB-HPM framework in detail. In the data acquisition layer, we developed crawler scripts to obtain Baidu street view panoramas, FANG.com house purchasing records and Baidu POI datasets. Before crawling the street view panoramas, it was necessary to implement road-simplifying procedures. In this article, we extracted the centerlines of the roads in Beijing and Shanghai and then obtained sample sites along the centerlines at 100-m intervals. The street view crawler exploited the Baidu static panorama API and obtained urban scene panoramas using multicore parallel computing by reading the coordinates of urban road sample points to save time. The implementation of the crawler was based on the methods in Dong et al. [51]. To reduce the influence of the different times when the street view pictures were captured on the empirical results and improve the comparability of the results between the two cities, only the panoramas that were captured between March and October 2015 were selected (we used the Baidu street view panorama IDs to extract the timestamps). Finally, we obtained 71,332 and 91,759 panoramas in Beijing and Shanghai, respectively. In addition, the housing price datasets were extracted from the FANG.com platform (one of China’s largest real estate transaction platforms). The Baidu POI data mainly included the point shapefiles of airport, bus, train and subway stations, parks, restaurants, hospitals, shops, and financial and educational facilities. The implementation of the POI data crawler was based on the methods in Xiao et al. [46]. Relevant source codes can be found at https://github.com/yonglinZ/article_codes.git.

PSPNet, which is a pixel-level semantic segmentation method, was employed in the data processing layer to parse massive street view pictures to obtain the GVI, BVI and SVI indicators (https://github.com/yonglinZ/pspnet_item.git). The location and neighborhood characteristics were calculated based on road distance using the Network Analyst tool in ArcGIS 10.2. The structure characteristics were included in the FANG housing price datasets and could be used directly.

In the modeling layer, we constructed HPMs using the four major characteristics (location, structure, neighborhood and scene perception characteristics) obtained from the previous layer as independent variables and the housing prices at the plot level as the dependent variable. Robust ordinary least squares (OLS) models were built using STATA 14 to finally generate the models, tests and statistical results. The STATA codes can be found in our GitHub repository; please see https://github.com/yonglinZ/article_codes.git.

### Using PSPNet to parse plot-level urban scene perception

In this study, we mainly focused on the three most important scene elements: greenery, sky openness and buildings. One scene perception element was defined as the percentage of pixels associated with the specific element to the total number of pixels in a Baidu street view panorama. This percentage represented the percentage of a visual element in a person’s normal field of view. For instance, the sky view index represents the percentage of sky pixels to the total number of pixels in a panorama. Therefore, the general formula (1) can be used to measure the appearance of scene perception elements.

$${VI}_{obj}=\frac{\sum_{i=1}^{n}{PIXEL}_{obj}}{\sum_{i=1}^{m}{PIXEL}_{total}}\times 100\%,obj\in \{greenery,building,sky\},$$ (1)

In formula (1), *VI*_*obj*_ represents the percentage of visual element *obj* in a panorama; $\sum_{i=1}^{m}{PIXEL}_{total}$ represents the total number of pixels in a panorama; and $\sum_{i=1}^{n}{PIXEL}_{obj}$ represents the number of pixels associated with visual element *obj*. For easy identification and understanding, the variables *VI*_greenery_, *VI*_building_ and *VI*_sky_ are referred to as GVI, BVI and SVI, respectively.

**Fig 3:**
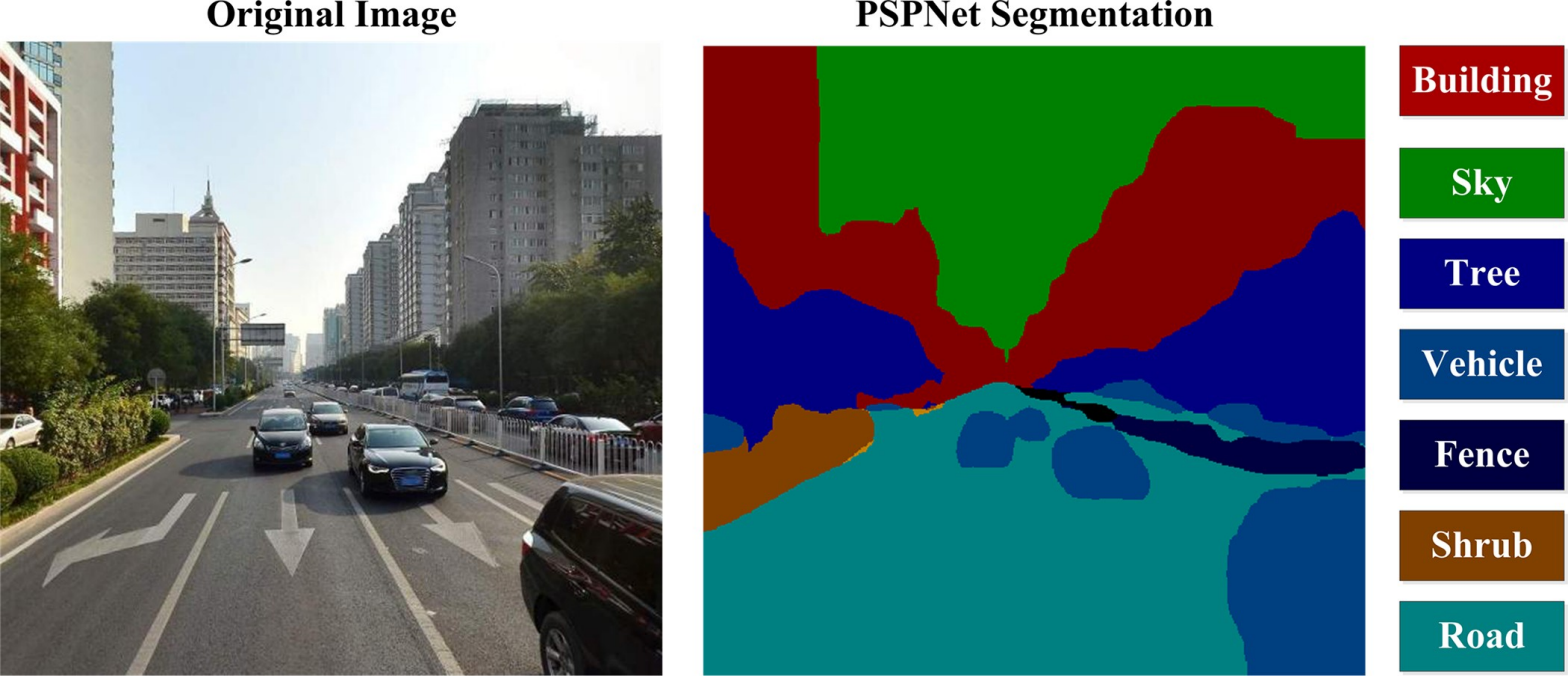
An example of PSPNet semantic segmentation (the image is an illustration and not from baidu street view to comply with copyright issues).

The scene parsing procedure involved object recognition and classification in massive panoramas. We adopted the PSPNet deep learning framework to parse urban scenes at the street level. A sample image and its segmentation results are shown in Fig 3. PSPNet is a pixel-level semantic segmentation method, and this framework made remarkable achievements in the ImageNet scene parsing challenge in 2016. The pixel-level accuracy of segmentation reached 93.4%, 92.9% and 95.4%. The PSPNet uses a pyramid pooling module to offer additional contextual information to reduce the probability of false segmentation; hence, it exhibits advantages in parsing complicated scenes with diverse visual elements. This article used the MIT ADE20K dataset [62] to pretrain the model. The hardware included two NVIDIA TITAN (12 GB memory storage) graphics cards, 64 GB physical memory, an Intel Xeon E5-2630 CPU (central processing unit), and two operating systems (Windows 10 professional and Ubuntu 14.04 LTS).

**Fig 4:**
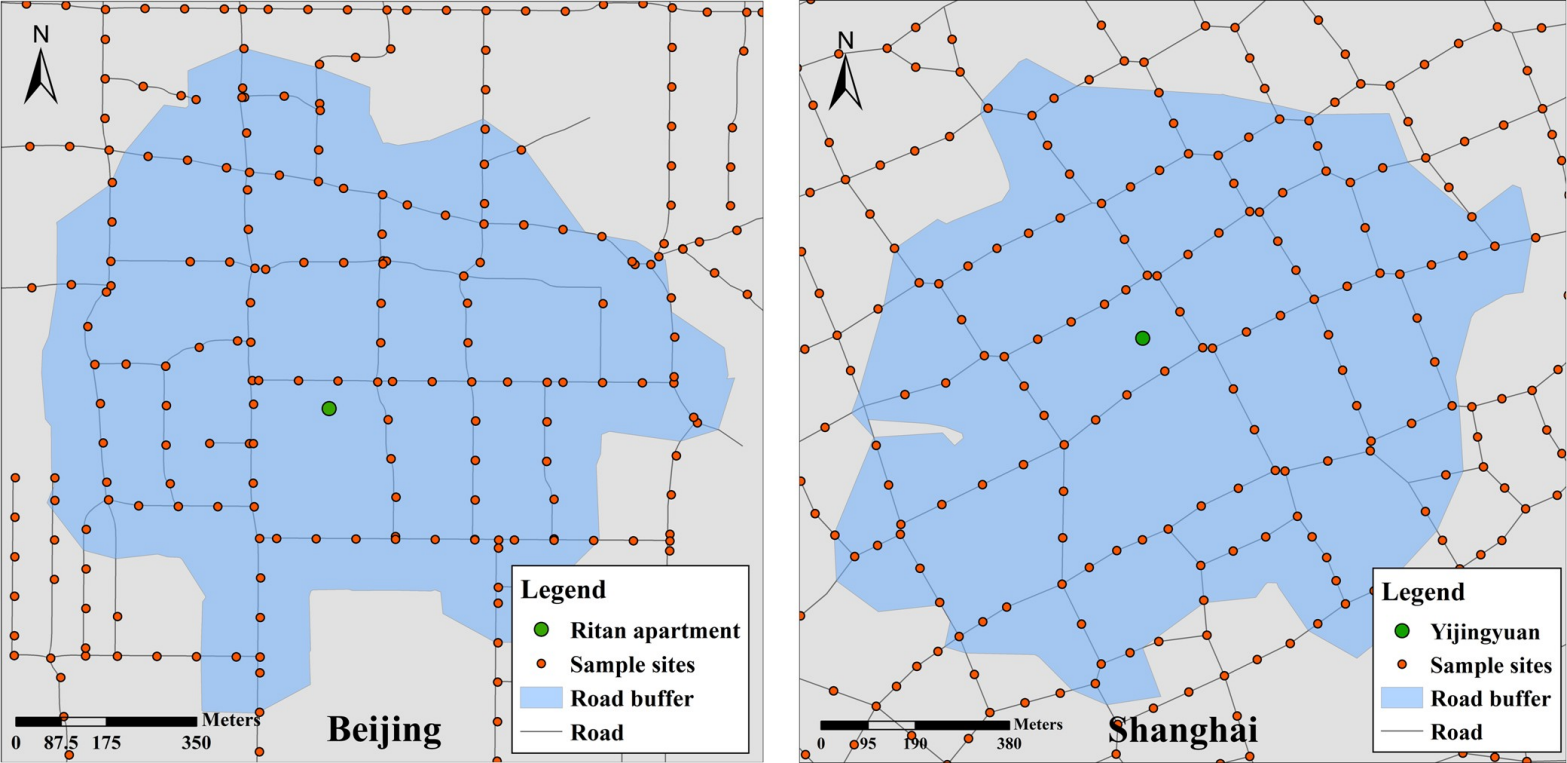
The road sites around two sample plots and their road buffer extents.

To quantify plot-level scene perception, we used the Network Analyst tool to build 800-m road buffers surrounding residential unit points based on the regular walking ranges of residents and our experience. The average GVI, BVI and SVI values within the road buffers were calculated and aggregated. The average numbers of road sample sites were 59 and 71 in Beijing and Shanghai, respectively. Taking the apartments in Ritan of Beijing and Yijingyuan of Shanghai as examples, Fig 4 shows the sample sites surrounding the housing plots and the road buffer extents.

### Hedonic price model and variable descriptions

The HPM has three common forms: linear models, semilog models and double-log models. In this study, the double-log form was adopted to build HPMs in Beijing and Shanghai because of the distribution of model variables. The GVI, BVI and SVI values at the residential unit level showed the characteristics of the lognormal distribution, and therefore, the indicators were subjected to natural logarithm transformation. Based on the basic theory of the HPM, the double-log form HPM can explain the percentage change in the dependent variable caused by each unit of change in one independent variable when the other independent variables are held constant. The HPM formula is given below.

$$lnPRICE=\alpha_{0}+\sum \beta_{\tau }C_{\tau }+lnGVI+lnBVI+lnSVI+\varepsilon ,$$ (2)

In formula 2, *PRICE* is the housing transaction price at the residential unit level; ln*PRICE* is the natural logarithm of *PRICE*; β_*τ*_ is the nonnormalized coefficient of each model variable, *τ*∈[1,2,3,…,21]; ln*GVI*, ln*BVI*, and ln*SVI* are the natural logarithms of GVI, BVI and SVI, respectively; α_0_ is the intercept term; and ε is the error term. The basic statistical information and descriptions of the variables are shown in Table 1. The data frames used to build the HPMs can be found at https://doi.org/10.6084/m9.figshare.7823975.v1.

**Table 1:** Variable descriptions and basic statistical information.

Cities		Beijing		Shanghai	
Variables	Description	Mean	Standard deviation	Mean	Standard deviation
*Dependent variable*					
LNPRICE	Log selling price in 10,000 RMB (Chinese currency, US $1 = RMB 6.71)	5.64	0.54	5.43	0.58
*Location characteristic*					
CENTER	Road distance to city center (km)	10.87	6.32	15.11	9.30
*Structure characteristics*					
AREA	Average usable area in the apartment (m^2^)	88.45	96.84	86.58	44.97
AGE	2018 minus the year of construction of the building	19.64	13.75	22.78	23.90
ORI	Dummy variable, 1 if the building has windows facing south	0.78	0.41	0.93	0.25
HS	Number of households	1030	1200	1037	942
FR	Floor-area ratio	2.52	1.55	2.27	1.61
PF	Property management fee (RMB/m^2^ per month)	1.76	1.42	1.27	1.28
GR	Green coverage rate (%)	0.32	0.07	0.34	0.11
BU	Dummy variable, 1 if the building is a slab; 0 if the building is any other type	0.60	0.49	0.87	0.34
*Neighborhood characteristics*					
AIRP	Road distance to the nearest airport (km)	22.13	6.75	13.56	8.58
BUS	Road distance to the nearest bus station (km)	0.24	0.20	0.18	0.16
SUB	Road distance to the nearest subway station entrance (km)	1.47	1.33	1.63	2.11
TRAIN	Road distance to the nearest train station (km)	4.74	3.04	7.09	5.26
FINAN	Road distance to the nearest financial facility (km)	0.26	0.36	0.32	0.36
RESTA	Road distance to the nearest restaurant (km)	0.14	0.21	0.15	0.22
HOSP	Road distance to the nearest hospital (km)	0.18	0.22	0.23	0.24
EDU	Road distance to the nearest educational facility (km)	0.18	0.24	0.21	0.24
SHOP	Road distance to the nearest shopping mall (km)	0.10	0.17	0.09	0.16
PARK	Road distance to the nearest park (km)	1.66	1.34	1.46	1.08
WATER	Road distance to the nearest water body (km)	0.73	0.50	0.35	0.28
WATER_A	Area of the nearest river or lake (km^2^)	0.12	0.22	0.11	0.28
*Scene perception characteristics*					
LNGVI	Logarithmic mean green view index within 800 m road distance	2.80	0.34	2.97	0.31
LNBVI	Logarithmic mean building view index within 800 m road distance	2.22	0.54	2.37	0.55
LNSVI	Logarithmic mean sky view index within 800 m road distance	3.74	0.16	3.65	0.26

The independent variables in this article were classified into four major categories: location, structure, neighborhood and scene perception characteristics. The location characteristic included one variable named CENTER, which represented the shortest road distance from a residential unit to the city center. To be more realistic, road distance and road network buffers were adopted for all distance calculations in this paper.

The structure characteristics included AREA (average usable area in the apartment), year (year of construction), HS (households), FR (floor-area ratio), PF (property management fee), GR (greening rate) and the dummy variables ORI and BU. The dummy variable ORI represented the main orientation of the buildings in the residential unit: if the buildings mainly face south, the value was set to 1; otherwise, it was set to 0. The dummy variable BU represented the major building type: if the building was a slab, the value was set to 1; otherwise, it was set to 0.

The neighborhood characteristics included the road distances between the housing estates and diverse urban amenities and environmental infrastructures, including the shortest road distances to airports (AIRP), bus stations (BUS), subway stations (SUB), train stations (TRAIN), financial facilities (FINAN), restaurants (RESTA), medical facilities (HOSP), educational facilities (EDU), commercial facilities (SHOP), urban parks (PARK), and water bodies (WATER), as well as the area of the nearest water body (WATER_A).

Finally, the newly introduced scene perception characteristics (GVI, BVI and SVI) at the residential unit level were transformed by the natural logarithm before building the HPMs. The coefficients of the independent variables can explain the percentage change in the dependent variables in the HPMs.

## Results and discussion

### Mapping scene perception indicators

**Fig 5:**
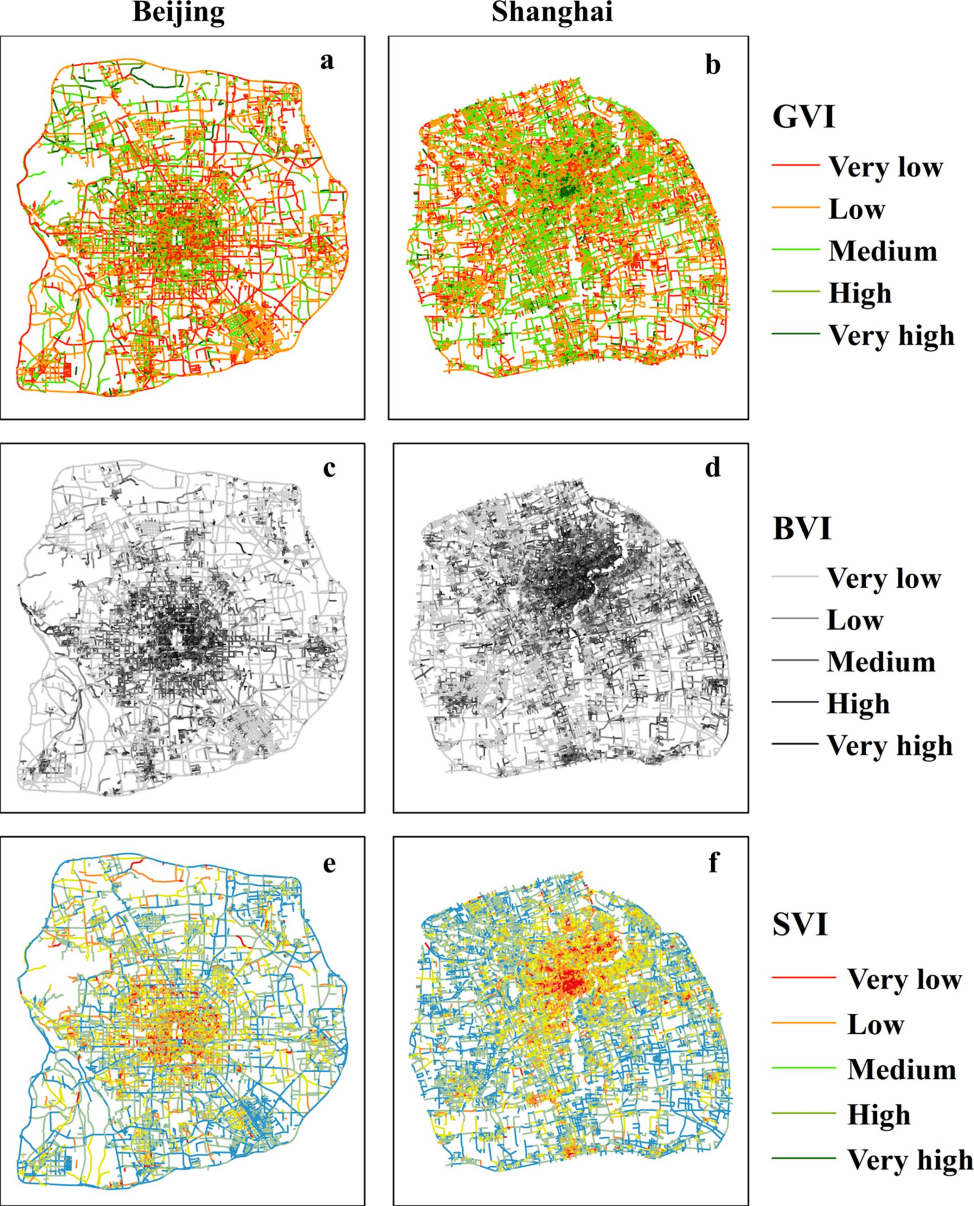
The spatial distributions of VIs in Beijing and Shanghai (GVI, BVI and SVI).

To improve the observations and comparisons, this study plotted the street-level GVI, BVI and SVI values in thematic maps for Beijing and Shanghai in 2015, as shown in Fig 5. The three view indexes (VIs) were each mapped using five value intervals, and the value intervals in Beijing and Shanghai were the same. The GVI value intervals were [0.00, 0.10], [0.11, 0.20], [0.21, 0.30], [0.31, 0.40], and [0.41, 0.70]; the BVI intervals were [0.00, 0.05], [0.06, 0.10], [0.11, 0.20], [0.21, 0.30], and [0.31, 0.65]; and the SVI intervals were [0.00, 0.20], [0.21, 0.35], [0.36, 0.45], [0.46, 0.55], and [0.56, 0.65], which correspond to the qualitative levels of very low, low, medium, high and very high.

The statistical results indicate that the average GVI values in Beijing and Shanghai are 16.44 and 19.49, respectively, which indicated that pedestrians in Shanghai tend to see more green scenes than those in Beijing. Fig 5(A) and 5(B) indicate that high and very high GVI road segments have a broader distribution and in general look “greener” in Shanghai than in Beijing. The GVI values of road segments above the high level are mainly located in the central areas (Dongcheng and Xicheng Districts), the northwestern area (Haidian District) and part of the southern area, but the high GVI segments are mainly concentrated along the western bank of the Huangpu River in Shanghai. The statistical results indicate that the average BVI value in Shanghai is slightly higher than that in Beijing (10.7 and 9.21, respectively). The spatial distributions in Fig 5(C) and 5(D) show that the BVI values in both cities present low-inside-and-high-outside gradient variations. The spatial distributions of the SVI values in Beijing and Shanghai show the opposite pattern relative to the BVI values (Fig 5(E) and 5(F)) and present high-inside-and-low-outside gradient variations. According to the statistical results, the average SVI of Beijing is higher than that of Shanghai (42.10 and 38.47, respectively). Therefore, from an overall perspective, people in Shanghai tend to see more profile areas of greenery and buildings, whereas people in Beijing tend to see more open road canyons.

### OLS regression modeling

The OLS regression modeling was four-fold: first, all independent variables were incorporated into the OLS model calculation; second, all VIs were removed from the model, but other variables remained the same; third, the GVI was added to the OLS model; fourth, the GVI and BVI were added to the model. The last three models controlled the VIs to show their contributions to the adjusted R^2^, and we included the last three models in the Supporting Information to show the soundness of our model (S1 File).

The model results in Table 2 show that the adjusted R^2^ values were 0.7242 and 0.7511 for Beijing and Shanghai, respectively, and most of the independent variables were statistically significant. The variance inflation factor (VIF) test was performed to verify whether the variables exhibited multicollinearity. The VIFs of the variables (less than 4) indicated that the multicollinearity effect was not significant in Model 1 or Model 2. In addition, the Durbin-Watson results were very close to 2, which indicated that the autocorrelation effect was not significant.

**Table 2:** OLS regression model (all variables).

		Model 1 (Beijing)			Model 2 (Shanghai)		
Variables	Full name	Unstandardized Coefficients	Standard Error	VIF	Unstandardized Coefficients	Standard Error	VIF
Constant	Constant	3.828***	0.4116		5.55***	0.2933	
*Location characteristic*							
CENTER	City center	-0.0452***	0.0012	2.51	-0.0154***	0.0012	3.45
*Structure characteristics*							
AREA	Housing area	0.0079***	0.0002	1.55	0.0099***	0.0001	1.39
AGE	Building age	-0.0027***	0.0001	1.19	-0.0001	0.0001	1.05
ORI	Orientation	0.0679***	0.0131	1.13	0.0532***	0.0204	1.02
HS	Households	0.0000	0.0000	1.36	0.0000	0.0000	1.49
FR	Floor-area ratio	-0.0084**	0.0034	1.18	0.0000	0.0000	1.01
PF	Property management fee	0.0324***	0.0054	1.47	0.0021	0.0028	1.13
GR	Green coverage rate	0.2421***	0.0822	1.18	0.2744***	0.0832	1.35
BU	Building types	0.0322***	0.0119	1.21	0.0189	0.0225	1.05
*Neighborhood characteristic*							
AIRP	Airport	0.0176***	0.0091	1.17	-0.0077***	0.0013	3.46
BUS	Bus station	-0.0117	0.0315	1.15	-0.0447	0.0392	1.14
SUB	Subway station	-0.0172***	0.0048	1.59	-0.0073*	0.0039	1.60
TRAIN	Train station	0.0024	0.0022	1.83	-0.0006	0.0013	1.88
FINAN	Financial facility	-0.0858***	0.0189	1.94	-0.0892***	0.0190	1.78
RESTA	Restaurant	0.1017***	0.0389	2.18	0.0421	0.0511	1.65
HOSP	Hospital	0.0151	0.0360	1.90	0.0116	0.04134	1.85
EDU	Educational facility	-0.0614*	0.0330	1.46	-0.0958***	0.0362	1.69
SHOP	Shopping mall	0.1149**	0.05178	2.69	0.1042	0.0677	2.70
PARK	Park	-0.0033	0.0048	1.62	-0.0153***	0.0059	1.40
WATER	Water body	0.0337***	0.0102	1.16	0.0416*	0.0246	1.22
WATER_A	Water body area	0.0447**	0.0215	1.12	0.0226	0.0367	1.08
*Scene perception characteristics*							
LNGVI	Green view index	0.2273***	0.0818	3.31	0.1853***	0.0471	2.71
LNBVI	Building view index	-0.0005	0.0018	3.96	-0.0008	0.0014	2.64
LNSVI	Sky view index	0.0899***	0.0280	3.17	0.0466***	0.0298	3.50
*F* ratio		200.5200			171.1500		
Adjusted *R*^2^		0.7242			0.7511		
Durbin-Watson		1.7628			1.8212		

* Indicates significance at the 10% level

** indicates significance at the 5% level

*** indicates significance at the 1% level.

Model 1 in Beijing showed that the location characteristic CENTER, the structure characteristics AREA, AGE, ORI, FR, PF, GR and BU, the neighborhood characteristics AIRP, SUB, FINAN, RESTA, WATER and WATER_A, and the scene perception characteristics LNGVI and LNSVI were significant at the 0.01 level. FR, SHOP and WATER_A were significant at the 0.05 level. The variable EDU was significant at the 0.1 level. The other independent variables were not significant in Model 1. Among the significant variables, CENTER, AGE, HS, FR, BUS, SUB, FINAN, EDU and PARK showed negative correlations; the other significant variables showed positive correlations.

Model 2 in Shanghai showed that the location characteristic CENTER, the structure characteristics ORI and GR, the neighborhood characteristics AIRP, FINAN, EDU, and PARK, and the scene perception characteristics LNGVI and LNSVI were significant at the 0.01 level. The variables SUB and WATER were significant at the 0.1 level. The other variables were not significant. Among the significant variables, CENTER, SUB, FINAN, EDU and PARK had negative correlations with the dependent variable, whereas the other significant variables had positive correlations. Therefore, in the scene perception characteristics, the newly included GVI and SVI values were significant in both megacities.

In Beijing, the coefficient of green view index in natural log (LNGVI) was 0.2273, which indicated that each 1% increase in green view index (GVI) was associated with a 0.2273% increase in housing price, indicating an elasticity of 0.2273. The monetary value of a 1% increase in GVI can be calculated based on the mean housing price and the elasticity, which was 39,377 RMB. Similarly, each 1% increase in sky view index (SVI) was associated with a 0.0899% increase (6011 RMB) in the plot-level average housing price. However, building view index in natural log (LNBVI) was not significant in Model 1.

In Shanghai, the coefficient of LNGVI was 0.1853, which indicated that each 1% increase in GVI was associated with a 0.1853% increase in housing price (21,689 RMB). Similarly, each 1% increase in SVI was associated with a 0.0466% (2763 RMB) increase in housing price. LNBVI was not significant in Model 2.

Moreover, the marginal prices of GVI in Beijing and Shanghai were compared, which indicated that the residents of Beijing showed stronger willingness to pay than the residents of Shanghai (39,377 and 21,689 RMB, respectively). The marginal price of SVI in Beijing was also larger than that in Shanghai (6011 and 2763 RMB, respectively). Therefore, the residents in the two cities exhibit obvious differences in their willingness to pay for greenery and sky openness, and the willingness was stronger in Beijing than Shanghai.

In the Supporting Information files (S1 File), we estimated three additional models to determine how much R^2^ increases when the scene perception characteristics were added in a stepwise fashion. In Beijing, the R^2^ contributions from GVI, BVI and SVI were 0.0214, 0.0032 and 0.0175, respectively; in Shanghai, the R^2^ contributions from GVI, BVI and SVI were 0.0184, 0.0025 and SVI was 0.0088, respectively. The three view indexes (VIs) increase R^2^ by 0.0421 and 0.0297 in Beijing and Shanghai, respectively, compared with the baseline models without them. The additional three models indicated that our results from Table 2 were sound. Therefore, greenery and sky openness around the housing estates were potential environmental amenities that buyers were willing to pay for and should be considered implicit factors in HPM studies.

### The impact of scene perceptions on housing price

Based on the state-of-the-art deep learning method, this article quantified several major scene perception indicators–GVI, BVI and SVI–and analyzed the impacts of scene perceptions on housing prices through the newly built OADB-HPM framework. The model results in the previous section showed that street-level GVI and SVI had positive effects on the neighboring housing prices in both Beijing and Shanghai, indicating that house buyers may have requirements for urban greenery and sky openness; however, BVI was not significant in any model, indicating that house buyers did not favor more visible artificial buildings in their living environment. According to the marginal price calculation, an increase in GVI and SVI by 1% can increase housing prices by 0.2273% and 0.0899%, respectively, in Beijing and by 0.1853% and 0.0466%, respectively, in Shanghai (the conversion formula for the marginal prices for the dummy logarithm transformed variables were e^*x*^−1, where *x* is the nonnormalized coefficient in the model). Therefore, the house buyers in Beijing and Shanghai favor the perception of natural aspects (green vegetation and open sky).

Through a comparison of the model results, home buyers in Beijing and Shanghai exhibited the same willingness to pay for the GVI and SVI, but there were significant differences in the marginal prices. Residents in Beijing showed significantly more willing to pay for GVI than those in Shanghai, which may be caused by the locations, climates and seasons of the two cities. Moreover, the two cities were located at different latitudes, and the green leaf period in Beijing is shorter than that in Shanghai due to climate, humidity and seasonal changes. This difference may be one of the major reasons affecting people's perception and willingness to pay for the greenery around housing estates.

In summary, according to the results in this article, the government should attach importance to the adjustment and optimization of the visual scenery around residential areas and high-rise building clusters to improve people’s visual access to natural elements. For example, the construction of open spaces and green parks in the public spaces between neighboring housing estates may help to meet the needs of people. The government can fine-tune and reshape the urban spatial structure according to the needs of residents to build an appropriate living environment to meet people’s practical needs. For instance, the configuration of three-dimensional green walls, corridors and roads can increase the daily interaction opportunities between people and green vegetation, and the appropriate use of domes made of transparent material and open walls may increase the sense of openness in various spaces.

The government should pay attention to the potential economic impacts of urban scene perception on real estate. On the one hand, in the absence of government control, real estate developers could exploit urban green and open spaces for additional benefits. Over time, the excessive accumulation of such benefits may lead to low-income consumers being unable to bear the financial costs resulting from increased housing prices. This phenomenon is detrimental to the sustainable development of the environment, society and economy. This phenomenon not only violates the fairness of the living environment in cities but also may harm the overall interests of citizens. On the other hand, in terms of the pleasant visual environment surrounding a property, developers almost unconditionally obtain the economic benefits of a pleasant residential environment but do not contribute to the maintenance or optimization of the urban visual landscape. Therefore, the government should collect real estate tax from real estate developers according to the constructions of urban scenes, and this revenue could be used to fund the optimization and maintenance of urban scenes. The government needs to strengthen the supervision of housing sales in the real estate market, restrain behaviors that abuse green scenes and open spaces as bargaining chips in the process of real estate sales to disrupt the market, and prevent developers from maliciously raising housing prices by citing the appropriateness of the public visual environment during the sales process. The goals of real estate developers should be to properly use the influence of urban visual elements on housing price on the basis of reasonable bidding and scientific planning, improve the competitiveness of their real estate brands, and participate in the construction of high-quality residential environment.

In summary, this article identified the actual requirements for visible greenery and sky openness in Beijing and Shanghai based on the newly constructed OADB-HPM framework. Due to the previous lack of technology and appropriate research framework, the potential effect of the visible element characteristics of residential environments on housing prices has often been ignored by researchers and decision makers. However, the framework and technical means proposed in this paper can now be used as an effective supplement and quantification tool.

### Limitations and future works

This study has opportunities for further improvement. Through pretraining, a more precise deep learning model could be generated for a better segmentation effect. In our future work, we will focus on more subtle visual characteristics in urban scenes, for instance, the texture of buildings, sculptures, and materials, and the esthetic degree of landscaping along the roads (overgrown with weeds or trimmed by gardening designs), and their effect on pedestrians’ perceptions. We believe that the quantification of subtle perception features can be used to further explore the perceptions, preferences and demands of citizens in public scenes and even the effects on the real estate economy. Furthermore, this work provides guidance for the rational allocation of resources. In terms of the data sources, the major Internet real estate transaction platforms include not only FANG.com but also Homelink and Anjuke. Further studies should aggregate multiple sources of housing price data to provide a more comprehensive analysis. In terms of modeling, subsequent studies should consider using multiple temporal and spatial HPMs in the analysis because people’s needs are likely to change dynamically over time. In addition, spatial econometric models can be used in subsequent studies to reduce model errors.

## Conclusions

This article used a state-of-the-art deep learning method to process massive street view panoramas to quantify the views of greenery, buildings and sky (GVI, BVI and SVI); then, these indicators were introduced into HPMs for the first time. This article mainly explored whether the newly included VIs affect the property-level housing prices in two typical Chinese megacities. We employed multisource open access datasets to quantify the locations and characteristics of residential neighborhoods. Taking the FANG.com housing price data as the dependent variable, HPMs were built and included four major types of influencing characteristics: location, structure, neighborhood and scene perception characteristics. The empirical results showed that the street-level GVI and SVI values in Beijing and Shanghai both have a positive effect on housing price, and the corresponding marginal implicit prices were RMB 39,377 and 21,689, respectively, in Beijing and RMB 6011 and 2763, respectively, in Shanghai.

## Supporting information

S1 File Additional experiments on hedonic price modeling. (DOCX)
