# Supplementary material for: Do street-level scene perceptions affect housing prices in Chinese megacities? An analysis using open access datasets and deep learning
Source: PLoS One. 2019 May 30;14(5):e0217505. doi: 10.1371/journal.pone.0217505 (PMC6542522; doi:10.1371/journal.pone.0217505)
Supplement: S1 File — (DOCX) [file pone.0217505.s001.docx]

# Supplementary information

# S1) Additional experiments on hedonic price modeling

## Contributions from the VIs (view indexes) to the models’ R^2^ values

We estimated the scene perception characteristics in the hedonic price model (HPM) to determine the contributions from the VIs to the model’s R^2^ values. The VI variables (GVI and BVI) were introduced into the standard HPM in a stepwise fashion with location, structure and neighborhood characteristics. Model S1 in S1 Table excluded all VI variables, Model S2 in S2 Table included only GVI, and Model S3 in S3 Table included GVI and BVI. The coefficients and significance of location, structure and neighborhood characteristics in Models S1, S2 and S3 were stable and sound in both study areas. In particular, the GVI, BVI and SVI in Models S1, S2, and S3 and the model in the main body of our paper (Table 2) were consistent in Beijing and Shanghai. The adjusted R^2^ values in the above four models showed that in Beijing, the R^2^ contributions from GVI, BVI and SVI were 0.0214, 0.0032 and 0.0175, respectively; in Shanghai, the R^2^ contributions from GVI, BVI and SVI were 0.0184, 0.0025 and 0.0088, respectively. Therefore, the visible-greenery and sky openness around the housing estates in Beijing and Shanghai were potential amenities that home buyers were willing to pay for and are potential implicit factors for HPM studies.

| **S1 Table. Model S1 (Without VI Variables).** | | | | | | | |
| --- | --- | --- | --- | --- | --- | --- | --- |
| **Road users** | **Beijing** | | |  | **Shanghai** | | |
|  | **Unstandardized Coefficients** | **Standard Error** | **VIF** |  | **Unstandardized Coefficients** | **Standard Error** | **VIF** |
| Constant | 4.9728*** | 0.0870 |  |  | 4.7697*** | 0.0535 |  |
| ***Location characteristic*** |  |  |  |  |  |  |  |
| CENTER | -0.0441*** | 0.0012 | 2.63 |  | -0.0160*** | 0.0012 | 3.45 |
| ***Structure characteristics*** |  |  |  |  |  |  |  |
| AREA | 0.0078*** | 0.0002 | 1.45 |  | 0.0097*** | 0.0005 | 1.46 |
| AGE | -0.0029*** | 0.0007 | 1.34 |  | 0.0000 | 0.0001 | 1.12 |
| ORI | 0.0699*** | 0.0132 | 1.23 |  | 0.0509** | 0.0206 | 1.20 |
| HS | 0.0000 | 0.0000 | 1.25 |  | 0.0000 | 0.0000 | 1.52 |
| FR | -0.0072** | 0.0034 | 1.27 |  | 0.0000 | 0.0000 | 1.09 |
| PF | 0.0331*** | 0.0054 | 1.53 |  | 0.0022 | 0.0030 | 1.17 |
| GR | 0.2261*** | 0.0844 | 1.22 |  | 0.2485*** | 0.0820 | 1.38 |
| BU | 0.0303*** | 0.0119 | 1.20 |  | 0.0251 | 0.0234 | 1.15 |
| ***Neighborhood***  ***characteristics*** |  |  |  |  |  |  |  |
| AIRP | 0.0175*** | 0.0009 | 1.21 |  | -0.0085*** | 0.0013 | 3.36 |
| BUS | -0.0140 | 0.0314 | 1.19 |  | -0.0335 | 0.0397 | 1.24 |
| SUB | -0.0189*** | 0.0047 | 1.46 |  | -0.0073* | 0.0040 | 1.56 |
| TRAIN | 0.0027 | 0.0022 | 1.88 |  | -0.0018 | 0.0013 | 1.90 |
| FINAN | -0.0911*** | 0.0192 | 2.014 |  | -0.0979*** | 0.0192 | 1.88 |
| RESTA | 0.1096*** | 0.0389 | 2.23 |  | 0.0269 | 0.0529 | 1.78 |
| HOSP | 0.0041 | 0.0357 | 2.01 |  | -0.0008 | 0.0416 | 1.88 |
| EDU | -0.0617* | 0.0332 | 1.59 |  | -0.0996*** | 0.0361 | 1.72 |
| SHOP | 0.1142** | 0.0517 | 2.46 |  | 0.1251 | 0.0701 | 2.78 |
| PARK | -0.0032 | 0.0047 | 1.42 |  | -0.0207*** | 0.0060 | 1.56 |
| WATER | 0.0304*** | 0.0105 | 1.19 |  | 0.0001* | 0.0000 | 1.25 |
| WATER_A | 0.0488*** | 0.0214 | 1.15 |  | -0.0255 | 0.0364 | 1.10 |
| *F* ratio | 247.6200 |  |  |  | 185.8700 |  |  |
| Adjusted *R*^2^ | 0.6821 |  |  |  | 0.7214 |  |  |
| Durbin-Watson | 1.8742 |  |  |  | 1.8823 |  |  |

| **S2 Table. Model S2 (Includes GVI).** | | | | | | | |
| --- | --- | --- | --- | --- | --- | --- | --- |
| **Road users** | **Beijing** | | |  | **Shanghai** | | |
|  | **Unstandardized Coefficients** | **Standard Error** | **VIF** |  | **Unstandardized Coefficients** | **Standard Error** | **VIF** |
| Constant | 4.8413*** | 0.0627 |  |  | 4.6805*** | 0.0890 |  |
| ***Location characteristic*** |  |  |  |  |  |  |  |
| CENTER | -0.0428*** | 0.0011 | 2.58 |  | -0.0160*** | 0.0012 | 3.45 |
| ***Structure characteristics*** |  |  |  |  |  |  |  |
| AREA | 0.0078*** | 0.0002 | 1.59 |  | 0.0097*** | 0.0005 | 1.39 |
| AGE | -0.0030*** | 0.0007 | 1.22 |  | 0.0000 | 0.0001 | 1.05 |
| ORI | 0.0707*** | 0.0132 | 1.21 |  | 0.0510*** | 0.0206 | 1.02 |
| HS | 0.0000 | 0.0000 | 1.30 |  | 0.0000 | 0.0000 | 1.49 |
| FR | -0.0076** | 0.0034 | 1.25 |  | 0.0000 | 0.0000 | 1.01 |
| PF | 0.0327*** | 0.0054 | 1.49 |  | 0.0022 | 0.0030 | 1.13 |
| GR | 0.2378*** | 0.0844 | 1.21 |  | 0.2499*** | 0.0819 | 1.35 |
| BU | 0.0309*** | 0.0119 | 1.21 |  | 0.0265 | 0.0234 | 1.05 |
| ***Neighborhood***  ***characteristics*** |  |  |  |  |  |  |  |
| AIRP | 0.0176*** | 0.0009 | 1.19 |  | -0.0084*** | 0.0013 | 3.46 |
| BUS | -0.0232 | 0.0318 | 1.15 |  | -0.0334 | 0.0397 | 1.14 |
| SUB | -0.0180*** | 0.0047 | 1.63 |  | -0.0070** | 0.0039 | 1.60 |
| TRAIN | 0.0033 | 0.0022 | 1.87 |  | -0.0018 | 0.0013 | 1.88 |
| FINAN | -0.0900*** | 0.0191 | 1.95 |  | -0.0955 | 0.0193 | 1.78 |
| RESTA | 0.1138*** | 0.0387 | 2.20 |  | 0.0262*** | 0.0528 | 1.65 |
| HOSP | 0.0108 | 0.0353 | 1.93 |  | 0.0006 | 0.0418 | 1.85 |
| EDU | -0.0593* | 0.0321 | 1.48 |  | -0.0957*** | 0.0368 | 1.69 |
| SHOP | 0.1137** | 0.0509 | 2.70 |  | 0.1212 | 0.0704 | 2.70 |
| PARK | -0.0025 | 0.0047 | 1.67 |  | -0.0202*** | 0.0060 | 1.40 |
| WATER | 0.0291*** | 0.0104 | 1.19 |  | 0.0001* | 0.0000 | 1.22 |
| WATER_A | 0.0471** | 0.0215 | 1.16 |  | -0.0226 | 0.0365 | 1.08 |
| ***Scene perception***  ***characteristics*** |  |  |  |  |  |  |  |
| LNGVI | 0.2461*** | 0.0149 | 2.42 |  | 0.1746*** | 0.0202 | 2.25 |
| *F* ratio | 236.0800 |  |  |  | 177.9300 |  |  |
| Adjusted *R*^2^ | 0.7035 |  |  |  | 0.7398 |  |  |
| Durbin-Watson | 1.8362 |  |  |  | 1.8825 |  |  |

| **S3 Table. Model S3 (Includes GVI and BVI)**. | | | | | | | |
| --- | --- | --- | --- | --- | --- | --- | --- |
| **Road users** | **Beijing** | | |  | **Shanghai** | | |
|  | **Unstandardized Coefficients** | **Standard Error** | **VIF** |  | **Unstandardized Coefficients** | **Standard Error** | **VIF** |
| Constant | 4.8413*** | 0.0627 |  |  | 4.5398*** | 0.1066 |  |
| ***Location characteristic*** |  |  |  |  |  |  |  |
| CENTER | -0.0428*** | 0.0011 | 2.58 |  | -0.0152*** | 0.0012 | 3.24 |
| ***Structure characteristics*** |  |  |  |  |  |  |  |
| AREA | 0.0078*** | 0.0002 | 1.59 |  | 0.0097*** | 0.0005 | 1.39 |
| AGE | -0.0030*** | 0.0007 | 1.22 |  | 0.0001 | 0.0001 | 1.05 |
| ORI | 0.0707*** | 0.0132 | 1.21 |  | 0.0543*** | 0.0205 | 1.02 |
| HS | 0.0000 | 0.0000 | 1.30 |  | 0.0000 | 0.0000 | 1.49 |
| FR | -0.0076** | 0.0034 | 1.25 |  | 0.0000 | 0.0000 | 1.01 |
| PF | 0.0327*** | 0.0054 | 1.49 |  | 0.0023 | 0.0030 | 1.13 |
| GR | 0.2378*** | 0.0844 | 1.21 |  | 0.6626*** | 0.0822 | 1.35 |
| BU | 0.0309*** | 0.0119 | 1.21 |  | 0.0279 | 0.0233 | 1.05 |
| ***Neighborhood***  ***characteristics*** |  |  |  |  |  |  |  |
| AIRP | 0.0176*** | 0.0009 | 1.19 |  | -0.0085*** | 0.0013 | 3.46 |
| BUS | -0.0232 | 0.0318 | 1.15 |  | -0.0373 | 0.0394 | 1.14 |
| SUB | -0.0180*** | 0.0047 | 1.63 |  | -0.0074* | 0.0039 | 1.60 |
| TRAIN | 0.0033 | 0.0022 | 1.87 |  | -0.0013 | 0.0014 | 1.88 |
| FINAN | -0.0900*** | 0.0191 | 1.95 |  | -0.0930*** | 0.0193 | 1.78 |
| RESTA | 0.1138*** | 0.0387 | 2.20 |  | 0.0331 | 0.0529 | 1.65 |
| HOSP | 0.0108 | 0.0353 | 1.93 |  | 0.0051 | 0.0416 | 1.85 |
| EDU | -0.0593* | 0.0321 | 1.48 |  | -0.0948*** | 0.0367 | 1.69 |
| SHOP | 0.1137** | 0.0509 | 2.70 |  | 0.1295 | 0.0707 | 2.70 |
| PARK | -0.0025 | 0.0047 | 1.67 |  | -0.0175*** | 0.0060 | 1.40 |
| WATER | 0.0291*** | 0.0104 | 1.19 |  | 0.0001* | 0.0000 | 1.22 |
| WATER_A | 0.0471** | 0.0215 | 1.16 |  | -0.0245 | 0.0365 | 1.08 |
| ***Scene perception***  ***characteristics*** |  |  |  |  |  |  |  |
| LNGVI | 0.246*** | 0.0149 | 2.42 |  | 0.1562*** | 0.0208 | 2.34 |
| LNBVI | -0.0337 | 0.0132 | 2.78 |  | -0.0360 | 0.0152 | 2.66 |
| *F* ratio | 236.0800 |  |  |  | 179.4600 |  |  |
| Adjusted *R*^2^ | 0.7067 |  |  |  | 0.7423 |  |  |
| Durbin-Watson | 1.7762 |  |  |  |  |  |  |
